# Exploring psychosocial experiences in clinically stable generalised Myasthenia Gravis: A thematic analysis

**DOI:** 10.1177/22143602251406948

**Published:** 2025-12-24

**Authors:** Ellie Maycock, Victoria Selby, Michael Hanna, Francesco Muntoni, Gita Ramdharry

**Affiliations:** 1 Institute of Neurology, University College London, London, UK; 2 Department of Psychology, University of York, York, UK; 3 Dubowitz Centre for Neuromuscular Diseases, Great Ormond Street Hospital, London, UK; 4 Institute of Child Health, University College London, London, UK; 5 National Hospital for Neurology and Neurosurgery, University College London Hospitals NHS Trust, London, UK

**Keywords:** myasthenia gravis, psychosocial, qualitative, thematic analysis, chronic illness, emotional wellbeing

## Abstract

Myasthenia Gravis (MG) is a chronic autoimmune neuromuscular condition that significantly impacts patients’ lives. Whilst psychosocial challenges are increasingly recognised as important in understanding the lived experience of patients, insight into these experiences during periods of clinical stability remains underexplored. This qualitative study explores the psychological and social aspects of living with MG through thematic analysis of semi-structured interviews with eight adults from a specialist MG care clinic in London, all considered clinically stable for at least three months. Data were derived from participants initially interviewed about their day-to-day experience of physical symptoms to inform development of a symptom monitoring tool. As psychosocial themes emerged strongly but remained unanalysed, this paper presents a secondary analysis focusing on psychosocial themes. Three overarching themes emerged: (1) *coping with adaptation* - including the burden of planning, struggles to accept diagnosis, therapeutic challenges and fear of the future; (2) *social and identity disruption* - involving changes in self-image, social withdrawal, and occupational challenges; and (3) *emotional and psychological impact* - highlighting negative emotions and cognitive fatigue. Uncertainty emerged as a meta-theme underpinning all other themes, reflecting, driving, and being created by MG's fluctuating symptoms. Findings suggest that psychological and social challenges underscored by uncertainty persist independently of symptom stability, highlighting the importance of uncertainty-focused interventions and integrated psychosocial support in MG care.

## Highlights

- Psychosocial challenges persist in well-managed myasthenia gravis cases.
- Patients face long-term adaptation, identity shifts, and emotional burden.
- Uncertainty and cognitive fatigue are central challenges in daily life.
- Qualitative data highlight overlooked care needs in stable MG populations.
- Findings support integrating psychological support into MG care pathways.

## Introduction

Over the past century, advancements in medical treatment have greatly improved the management and prognosis of Myasthenia Gravis (MG), allowing many patients to achieve substantial control of their physical symptoms or partial remission.^1^ While these treatments have significantly enhanced daily functioning for most individuals with MG, residual issues such as intermittent muscle weakness, fatigue, and mild neuromuscular symptoms can persist, even in well-managed cases.^2^ Fatigue remains particularly pervasive, driven by both physical muscle weakness and fatigability inherent to MG, and cognitive fatigue, often described as “brain fog”.^3^ These symptoms often fluctuate, particularly following physical exertion or during periods of stress, adding unpredictability to daily life. The process of diagnosis and treatment can also be complex and emotionally taxing, with some patients experiencing traumatic events, such as severe respiratory crises, as part of their medical journey.^4^

Beyond the physical challenges, psychological and social factors have increasingly emerged in recent research as critical influences on the quality of life for individuals with MG. These impacts have been identified by patients as key priorities for further research and care planning.^5^ While earlier studies assumed that addressing physical symptoms would alleviate psychosocial difficulties,^6^ recent evidence suggests that these impacts persist even when stability of symptoms have been reached, and can affect well-being independently of symptom control.^7^ Despite calls for a more integrated biopsychosocial approach to MG to provide holistic support and a recent increase in research examining patient experience of the psychosocial dimensions of MG, these remain underrepresented in the literature and inadequately addressed in clinical practice.^8^

Mental health issues are particularly prevalent in MG, with patients experiencing anxiety and depression at higher rates than the general population.^9^ However, recommendations for screening and treating psychiatric comorbidities in MG remain scarce and often inconsistent.^10^ Persistent social and emotional difficulties - such as unemployment^11^ relationship strain,^12^ stigma,^5^ and issues with sleep^13^ and memory^14^ - further compound these issues, even for patients with minimal manifestations of physical symptoms.

Phenomenological research by Keer-Keer et al. exploring the lived experiences of adults with MG underscores the profound psychosocial impact of the condition's chronic and unpredictable nature, which disrupts patients’ sense of time, space, body, and relationships.^15^ Law and colleagues^7^ identified patient-led themes that characterise the experience of living with MG, including frequent frustration due to fluctuating symptoms and treatment inertia, which impose limitations on daily activities, necessitate continual trade-offs, and contribute to negative self-perception and emotions. Moreover, Jackson et al.^5^ found that all patients they interviewed reported both negative emotional and practical impacts on their work and finances. These issues, exacerbated by stress, may contribute to physical symptom exacerbations, further underscoring the need for holistic care approaches.^16^

However, few studies have specifically examined the persistent psychosocial challenges faced by individuals whose physical symptoms have remained stable for a period of time.^5,7^ Understanding these challenges, along with the specific life domains they affect and their underlying factors, could inform new psychotherapeutic strategies to improve quality of life.

This study presents an exploratory thematic analysis of the psychosocial impacts experienced by individuals diagnosed with MG whose responsible clinician has deemed them clinically stable for a minimum of 3 months without symptom fluctuation. Drawing on in-depth qualitative interviews, it explores the psychological and social concerns that persist despite reasonably stable physical symptom profiles. The findings highlight potential interventions to enhance quality of life and emphasise the importance of addressing psychosocial functioning in MG care.

## Methods

### Study design

This study is a qualitative secondary analysis of data, focusing on individuals with MG. The dataset was originally generated as part of a broader study exploring daily experiences of both MG and congenital myasthenic syndromes (CMS). That primary study aimed to identify residual physical challenges, particularly difficulties with activities of daily living, to inform the development of a home-based symptom-monitoring tool.^17^ Whilst not the focus of the original study, psychological and social themes were identified in the broader dataset and were a recurrent theme characterising participants’ lived experience. This warranted more in-depth focus, which was not addressed by the primary research. This secondary analysis focuses specifically on the MG group, as individuals with MG have different disease progression patterns and subsequently likely somewhat distinct psychosocial experiences that are not directly comparable to those with CMS.

The decision to conduct a secondary analysis was motivated by the opportunity to maximise the utility of an existing dataset, given the rarity of MG, and to explore unexamined psychosocial themes without placing additional burden on participants. Following the recommendations of Ruggiano and Perry,^18^ we ensured transparency and rigour throughout the process by maintaining ethical standards such as data de-identification and researcher reflexivity, involving members of the original research team, analysing uncoded transcripts, and acknowledging the inherent limitations of this approach.

### Participant recruitment and sample

Participants were recruited purposively from the Myasthenia Outpatient Clinic at the National Hospital for Neurology and Neurosurgery. Adults with a confirmed diagnosis of generalised MG were invited to take part if they met the following criteria as confirmed by their responsible clinician: regular attendance at a specialist neuromuscular clinic, sufficient communication ability to participate in an interview, and clinical stability. Clinical stability was defined as no major symptom fluctuation, no hospital admission or treatment change for ≥3 months prior to interview. While longer thresholds (e.g., 6–12 months) are sometimes used in MG research, a 3-month period was chosen in line with prior MG research involving stable-disease cohorts (e.g., in COVID-19 vaccine safety studies^19^), and allowed feasible recruitment for this qualitative study.

Diagnostic confirmation was established by the treating neurologist using standard clinical and laboratory criteria including clinical presentation consistent with MG, positive response to anticholinesterase medication, and/or positive antibody testing (acetylcholine receptor or muscle-specific kinase antibodies).

For the purposes of this secondary analysis, we focused on the eight participants with comprehensive clinical information available. This decision was made to strengthen the clinical validity of our findings by situating participants’ psychosocial experiences within the context of their clinical profile. In addition to antibody status, data were available on current treatments, surgical history, comorbidities, and mental health diagnoses. Restricting the analysis to this subgroup enabled us to draw more meaningful links between lived experience and clinical course, which would have been difficult to achieve with incomplete data**.** Although diagnostic confirmation was permitted via clinical presentation and/or antibody positivity, in this sample all eight participants were seropositive. All participants included had generalised MG, rather than purely ocular disease, further enhancing the comparability of experiences. Table 1 summarises the individual-level and demographic and clinical characteristics of the group.

**Table 1.** Summary of demographic and clinical characteristics of participants with myasthenia Gravis (N = 8).

ID	Gender	Age (years)	Years since Dx	Antibody status	Current MG therapies	Comorbidities	Mental health treatments	Current areas of weakness
P1	F	30	2	MuSK+	Prednisolone, Mycophenolate	None	None	Diplopia, dysarthria, dysphagia, facial
P2	F	20	6	AChR+	Pyridostigmine, Thymectomy (2011)	None	None	Ocular, bulbar, fatigue
P3	M	70	5	AChR+	Pyridostigmine	Essential tremor, BPH	None	Ocular
P4	M	46	6	AChR+	Azathioprine, Thymectomy (2009)	Depression, hip pain, tracheostomy (2008)	None	Generalised
P5	F	56	3	AChR+	Prednisolone, Mycophenolate, Thymectomy (2011)	Depression, IBS	Sertraline	Generalised
P6	F	33	11	AChR+	Prednisolone, IvIg, Thymectomy (2003)	Optic atrophy (post-RTA), transient weakness	None	Dysarthria, dysphagia, ptosis, facial
P7	M	56	1	AChR+	Azathioprine, Thymectomy (2013)	Depression	CBT	Dysarthria, dysphagia, facial/bulbar, ptosis
P8	F	43	20	AChR+	Prednisolone, Pyridostigmine, Thymectomy (2004)	Hypothyroidism, PID, urticaria	None	Generalised, ophthalmoplegia, diplopia, bulbar
Group summary	5F (62.5%), 3 M (37.5%)	Mean 44.3 (SD = 16.5), range 20–70	Mean 6.8 (SD = 6.3), range 1–20	AChR + 7 (87.5%), MuSK + 1 (12.5%)	Prednisolone 4 (50%), Azathioprine 2 (25%), Mycophenolate 2 (25%), Pyridostigmine 3 (37.5%), IvIg 1 (12.5%); Thymectomy 6 (75%)	Depression 3 (37.5%), Essential tremor 1 (12.5%), IBS 1 (12.5%), Hypothyroidism 1 (12.5%), BPH 1 (12.5%), PID 1 (12.5%), Urticaria 1 (12.5%)	Sertraline 1 (12.5%), CBT 1 (12.5%)	Bulbar involvement 6 (75%), Ocular 5 (62.5%), Generalised 4 (50%), Fatigue 2 (25%)

Abbreviations: Dx = Diagnosis; Tx = Treatment; BPH = Benign Prostate Hypertrophy; IBS = Irritable Bowel Syndrome; RTA = Road Traffic Accident; PID = Pelvic Inflammatory Disease; Ivlg = Intravenous immunoglobulin.

### Data collection

Semi-structured, face-to-face interviews were conducted by a trained researcher (VS) with experience in qualitative research methods and a background in physiotherapy and neuromuscular conditions. The interviewer had received specific training in conducting research interviews and had prior experience working with patients with neurological conditions. Each interview lasted between 30 and 45 min, was transcribed verbatim, anonymised, and assigned a unique participant ID (P1-P8). The project was approved by the Research Ethics Committee (REC reference 14/EE/0033, NRES Committee East of England – Cambridge South), and all participants consented to secondary data use.

### Data analysis

Data were analysed using thematic analysis, following Braun and Clarke's six-phase framework.^20^ Two researchers were involved in the coding process. EM first familiarised herself with the transcripts through repeated reading, before undertaking line-by-line coding focused on psychological and social impacts of living with MG. Codes were then collated into preliminary themes, which were iteratively reviewed, refined, and defined to ensure analytic clarity.

To enhance rigour, intercoder reliability was established through independent coding of 20% of transcripts by a second researcher (GR). Coding discrepancies were discussed and resolved, leading to refinement of the coding framework. Regular discussions with the wider research team (MH, FM, GR) provided further validation of emerging themes, and consultation with clinical specialists ensured accurate representation of patient experiences.

NVivo 12 Plus software was used to facilitate systematic coding and theme organisation. Although the original study primarily focused on physical symptom experiences, sufficient breadth and depth were available for secondary exploration. In the present analysis, with a specific focus on psychosocial themes, no new codes emerged after the seventh interview, suggesting that thematic saturation was achieved in relation to this analytic focus, however we acknowledge that the small sample size may limit the diversity of perspectives captured, and future studies with larger and more varied cohorts are needed. The primary analyst (EM) was not involved in the original data collection and was blinded to participants’ physical symptom profiles during psychosocial coding, reducing potential bias and supporting the credibility of the findings.

## Results

Three overarching themes emerged from the thematic analysis, each encompassing sub-themes related to the psychosocial impacts of living with MG. All themes were related to a pervasive sense of uncertainty arising from the unpredictable nature of MG symptoms and the complex journey through diagnosis and treatment. This uncertainty acted as a cross-cutting meta-theme that influenced participants’ adaptation strategies, social functioning, and emotional wellbeing.

Themes and subthemes, along with an indication of how many participants referred to each, are presented in Table 2. Table 2 includes the number of participants contributing to each theme to illustrate the breadth of representation across the sample. These frequencies are not intended to suggest that more commonly reported themes carry greater importance. Theme development followed a qualitative, meaning-focused analytic approach, prioritising conceptual richness over frequency**.** Below, we describe each theme in turn, supported by illustrative quotations from participants that include participant demographics.

**Table 2.** Summary of themes and prevalence.

Theme	Subthemes	Participants mentioning
1. Coping with Adaptation		**8/8 (100%)**
	1.1 Burden of planning	8/8 (100%)
	1.2 Struggles to accept diagnosis	3/8 (37.5%)
	1.3 Therapeutic challenges	6/8 (75%)
	1.4 Fear of the future	3/8 (37.5%)
2. Social and Identity Disruption		**7/8 (87.5%)**
	2.1 Redefining self and social image	5/8 (62.5%)
	2.2 Social withdrawal and isolation	8/8 (100%)
	2.3 Occupational and functional challenges	7/8 (87.5%)
3. Emotional and Psychological Impact		**8/8 (100%)**
	3.1 Emotional distress	8/8 (100%)
	3.2 Cognitive fatigue	4/8 (50%)

### Coping with adaptation

A core psychological burden for all participants was the ongoing need to adapt to the demands of living with MG. This adaptation was necessary across physical, emotional, and practical aspects of their lives and was fundamentally shaped by the uncertainty inherent in the condition's unpredictable course.

#### Subtheme 1.1: burden of planning

A major sub-theme identified by all participants was the burden of managing energy through careful planning. The unpredictable and fluctuating nature of MG symptoms, even when reasonably stable, required participants to constantly monitor and conserve their energy to prevent worsening muscle weakness and symptom exacerbation.

“I plan everything. I’m not aware of it anymore. I don’t do anything on the spur of the moment really… It's like you’re saving up energy. Like, when I started my course, I had to stop swimming because I knew I couldn’t do both” (P5, Female, 56)

“I tend to plan my life carefully… Initially I thought I’ve got myasthenia it's fine, you know, get on with it, do what I want to do. But actually by not planning I became very depressed. I couldn't do what I really wanted to be able to do and that's what pushed me over the edge.” (P1, Female, 30)

This ‘energy negotiation’ involved making trade-offs between daily activities, with participants needing to prioritise important tasks and often cancelling other plans or engaging in more sedentary behaviours to maintain their strength. Different strategies were employed to manage this, ranging from keeping detailed diaries to reserving specific days for rest. Despite variations in approach, planning was described by all participants as an essential coping strategy, though it also imposed a significant cognitive and emotional toll. Many participants expressed feeling overwhelmed by the constant need to evaluate their energy levels and adjust their activities accordingly, with several noting feelings of disappointment or frustration when they were forced to cancel plans or limit their engagement in more active pursuits.

#### Subtheme 1.2: struggles to accept diagnosis

In addition to the physical demands of planning, three participants also described the emotional journey of adapting to and accepting their diagnosis as an enduring psychological challenge that extended well beyond the initial diagnostic period..

“I am very much in denial about myasthenia… There have been moments, glimpses when I’ve thought it's all over and this would go away” (P8, Female, 43)

“[My diagnosis is] not something that I have discussed in long detail, no… Not that I'm ashamed of it but I guess you don't want to feel disabled by making a big thing of accepting it. It feels like that diagnosis might define me and I don't want that so it's not something I focus on.” (P4, Male, 46)

Some participants described the continued hope that MG would ‘go away’ as a barrier to accepting their condition. The chronic nature of their condition was difficult to accept, and participants described this as a deeply personal, often lonely process. Notably, several participants indicated that they had not received formal support for this emotional adjustment and were left to manage thoughts like “why me?” on their own.

#### Subtheme 1.3: therapeutic challenges

Six participants reported significant difficulties navigating both treatment side effects and the healthcare system itself, which compounded their sense of uncertainty about their condition.

“I am just pyridostigmine and steroids and I'm not on the immunosuppressants. I was for a little bit but I came off as I felt terrible… I don't [take my medication 5 times a day]. Look I've tried everything, I've tried very hard to be compliant.” (P8, Female, 43)

“I think the care is really fragmented and scattered, I don't understand it… it started from diagnosis which took 1 year. I wasn't diagnosed, diagnosed different diseases, infection, stroke, antibiotics, then said it was psychological. If not find anything they say psychological.” (P6, Female, 33)

Participants described the difficulties of adjusting to new treatments, with aversive side effects such as constipation, weight gain, and flu-like symptoms being commonly associated with immunosuppressant medication. These side effects were often distressing and disruptive, leading some participants to struggle with adherence. A few participants explicitly mentioned discontinuing or avoiding treatments due to these challenges, further complicating their care. Participants shared experiences of a difficult and often prolonged journey to diagnosis, with many initially misdiagnosed (commonly with psychiatric conditions) before receiving an accurate diagnosis of MG. This misdiagnosis delayed access to effective treatment, which, for some, fostered an ongoing distrust of the healthcare system. A few participants noted that this was exacerbated by experiences of fragmented care, such as inconsistent treatment teams, or abrupt changes in their care plans. These disruptions made it difficult for participants to establish a stable and trusting therapeutic relationship, adding to their emotional burden. The unpredictable nature of their interactions with healthcare professionals mirrored the unpredictability of their symptoms, leaving participants feeling vulnerable and uncertain. Several participants expressed frustration at having to adapt to unreliable care delivery, which reinforced a broader sense of instability in managing their condition.

#### Subtheme 1.4: fear of the future

The uncertainty of the future was another source of psychological burden mentioned by three participants. They described having concerns about how their illness may unfold in the future and the further adaptations that may be required of them, worrying both about whether they would experience a deterioration in their symptoms, or concerned that their condition would not continue to improve and they may be ‘stuck’ at the level of impairment they were currently at.

“[The hardest thing about Myasthenia is] thinking about your future and like, is it going to improve or am I going to be like this forever? Can I achieve my goals and can I go out and do the things I want to do?” (P2, Female, 20)

“They said you’re going to get worse before you get better but we need to know where you are now, so that was just it.” (P4, Male, 46)

Participants reported fearing the potential loss of further abilities and had to confront the possibility of futures they struggled to imagine. The unpredictability of MG meant that participants were constantly questioning what further adaptations might be required. This uncertainty made it difficult for them to fully adjust to their condition, as the potential for future decline overshadowed their day-to-day experiences.

### Social and identity disruption

Seven participants reported significant disruptions to both social functioning and their sense of identity. The visible symptoms of MG, combined with its unpredictable nature, affected how participants saw themselves and how they believed others perceived them. These changes extended to social interactions, daily functioning, and occupational roles.

#### Subtheme 2.1: redefining self and social image

Five participants described feeling fundamentally changed by their diagnosis, struggling to reconcile their pre- and post-diagnosis identities. Participants described feeling like different people compared to before their diagnosis, with previously outgoing or spontaneous individuals becoming more withdrawn due to their illness. This shift in self-image left some questioning which version of themselves, the “before” or “after” diagnosis, was their true identity.

“I would have said before that I am probably an outgoing person, but some people might think I'm not now because sometimes the condition makes me feel reserved… I used to think of myself as ‘the crazy one’.. but not anymore.” (P2, Female, 20)

Social stigma exacerbated this struggle, as some participants worried that symptoms such as unsteadiness might be misinterpreted as being intoxicated, or needing to cancel plans leading others to believe they were ‘lazy’ or ‘unfriendly’. Additionally, communication difficulties arising from their difficulties with facial expressions led to concern about misunderstandings like people thinking they were less happy than they really were:

“The main thing at the moment is my speech and my face sometimes like, sometimes my housemate will be like smile, but I can't help it my face will just frown. That's how it is.” (P1, Female, 30)

The constant need to renegotiate their identity in social contexts added to their emotional burden and sense of isolation.

#### Subtheme 2.2: social withdrawal and isolation

All participants also discussed social withdrawal and isolation as a significant impact of living with MG. The unpredictable nature of their symptoms often led them to cancel social plans at the last minute or avoid making commitments altogether, out of fear they wouldn’t be able to follow through. This withdrawal had a direct impact on personal relationships, with several participants noting that they had fewer close friends as a result. One participant shared how MG symptoms contributed to the breakdown of a significant romantic relationship, while others spoke about feeling increasingly disconnected from their social circles.

“I've had periods where this has affected my social life, especially…I feel like it's why I don't have a partner. In fact when this came on, the last partner I had kind of ran away.” (P8, Female, 43)

“When I'm feeling like I am now, bad symptoms, I just email everyone to say sorry you won't see me until next week, I have to cancel.” (P5, Female, 56)

Participants reported this increase in isolation exacerbated feelings of loneliness, and reinforced anxieties about future socialisation.

#### Subtheme 2.3: occupational and functional challenges

Occupational and functional challenges further contributed to participants’ disruption of self-identity. Seven participants had experienced significant impacts on their work and career aspirations, with many forced to abandon professional goals or adapt their working lives entirely.

“The plan wasn't to get myasthenia, what I wanted to do was I wanted to do stand-up comedy. Basically I'm a qualified lawyer and I had the opportunity to take voluntary redundancy, and so what I wanted to do was to do something kind of completely different… So I wanted to start stand-up comedy and I did a little bit, but then I didn't really feel able to do anymore, I mean I'm pretty sure now I wouldn't be able to do a fulltime job.” (P7, Male, 56)

Beyond employment, participants described the loss of basic functional activities that had previously defined their independence and lifestyle:

“I used love walking. And working in Brighton it was actually a quicker way of getting around than bus, but that all came to, I wouldn't say a grinding halt but a gradual decline in terms of that.” (P3, Male, 70)

These limitations left participants grappling with a sense of loss, as they struggled to reconcile the gap between what they had previously enjoyed doing, and had envisioned for their lives with what they were now physically capable of achieving.

### Emotional and psychological impact

All participants described persistent challenges managing both emotional distress and cognitive difficulties that were central to their experience of living with MG.

#### Subtheme 3.1: emotional distress

The emotional toll of living with MG was mentioned by all the participants. Emotional distress manifested as four primary responses: frustration, embarrassment, anxiety, and low mood, often occurring in response to the unpredictability and limitations imposed by MG. Frustration was a frequent daily experience, arising as a response to the limitations imposed by MG. Many participants expressed feelings of helplessness when symptoms worsened unexpectedly or prevented them from participating in activities they enjoyed.

“It's more the physical side of things that really cause me a degree of frustration, no pain, just frustration because I used to be able to do it and now I can't now.” (P3, Male, 70)

Embarrassment was another common theme, with several participants describing how visible symptoms, such as falling or sweating in public, made them self-conscious. A few participants also mentioned feeling embarrassed when they had to cancel plans or couldn't achieve personal goals due to their symptoms.

“When I was walking on the bus one day… there was a very low step and my legs just buckled and I just fell… It was really embarrassing. I just went home to my mum and felt sad about it. Just thought ‘Help me’.” (P2, Female, 20)

Low mood and depression were also discussed. Some participants framed their emotional difficulties as an understandable reaction to the profound changes that MG had brought to their lives. While only one participant explicitly mentioned taking antidepressants, several described low mood as an almost inevitable consequence of the illness, given its ongoing psychological toll. Three participants reported a formal diagnosis of depression, and two had sought professional mental health support. As one participant explained:

“I’m on antidepressants now as well, I’ve been really suffering from depression but my counsellor said that if she’d gone through what I’d been through, she would too.’ (P5, Female, 56)”

Anxiety was a prominent concern for many participants, particularly among those who had experienced respiratory crises. The memory of these episodes often triggered ongoing fear of future relapses. Participants described the unpredictability of symptoms as intensifying this anxiety, leaving them in a state of heightened vigilance and uncertainty about what the future might hold. For some, this manifested as a persistent fear of potential triggers.

“I was quite depressed when I was sick and I went through a lot of years when I didn’t really care if I lived or died. But that only lasted for a very short time… I became very very scared… I guess it's because of the mycophenolate I’ve had 5 colds this year. I don’t kiss or hug people anymore. All those things I don’t do anymore because I’m scared of getting something.” (P5, Female, 56)

#### Subtheme 3.2: cognitive fatigue

Four participants experienced cognitive difficulties distinct from physical exhaustion, describing mental fog, concentration problems, and memory issues.

“Just exhaustion, mental exhaustion, I used to be really sort of vibrant mentally now it's like a fog. And I don't know if it's because of my eyes, not being able to pay attention to words or whether it's just myasthenia, I have no idea. But maybe it's the myasthenia because it's almost like I can't, I have a terrible memory and I don't think very logically anymore… And people think they are crazy because they can't think.” (P8, Female, 43)

“[Early warning sign of feeling worse is…] chronic fatigue… like an inner tiredness and it's inside and creeps out.” (P5, Female, 56)

This cognitive strain was particularly distressing, as it undermined participants’ confidence in their mental faculties and affected their sense of identity as capable, thinking individuals.

## Discussion

This thematic analysis suggests that psychosocial difficulties persist even among individuals whose MG symptoms have remained clinically stable for at least three months, with uncertainty emerging as a central meta-theme around which other challenges cluster. Three interconnected themes were identified across adaptive, social, and psychological domains, each with sub-themes that both align with and extend previous qualitative research on the lived experience of MG, offering new insights into the challenges faced during periods of stability.

The first theme, coping with adaptation, captured the ongoing demands of managing energy trade-offs, navigating diagnosis and healthcare, and confronting the uncertainty of a fluctuating condition. The second theme, social and identity disruption, described the impact of MG on self-concept, social withdrawal, and occupational functioning. The third theme, emotional and psychological impact, highlighted experiences of low mood, anxiety, and cognitive fatigue, often referred to by participants as “brain fog.” These domains were closely interconnected: difficulties in one area often exacerbated others, such as the burden of energy management contributing to social withdrawal.

Crucially, these experiences were also shaped by a persistent sense of uncertainty, understandable given the nature of a disease marked by fluctuating symptoms, fatigable muscle weakness, and often a prolonged diagnostic journey. Most themes were consistently reported across participants, regardless of age or illness duration, underscoring the widespread and enduring psychosocial impacts of MG. This finding challenges the notion that psychological and social difficulties are responses to symptom exacerbation and instead highlights the need for sustained psychosocial support even during periods of apparent medical remission.

### Coping with adaptation

A clear finding was that participants’ daily experiences were shaped by a constant need to make trade-offs between activities in order to conserve limited energy. This reflects the findings of Jackson et al.,^5^ who reported that individuals with generalised MG often struggled to engage in hobbies or sports, and that maintaining participation in everyday life required extensive planning. This finding is further supported by the large-scale study by Camdessanché et al.,^21^ which surveyed 255 French adults with generalised MG about their unfulfilled aspirations. Physical activity limitations dominated their findings, with sport, greater mobility, and reduced fatigue representing the most frequently cited unfulfilled aspirations. Similarly, Law et al.^7^ identified a continuous assessment and trade-off process as a fundamental characteristic of living with MG, describing how patients must navigate constant adaptation and continuous mental calculations about activity choices and energy management.

Our findings support these previous studies and suggest this cognitive burden persists even during periods of symptom stability, representing an enduring feature of the MG experience rather than a temporary response to active disease progression. The convergence of findings across different populations, sample sizes, and methodologies provides robust evidence that adaptation challenges are consistent, cross-cultural features of the MG experience.

Participants also described ongoing struggles to accept both the therapeutic demands of MG and the chronic, unpredictable course of the disease. The therapeutic challenges reported by 75% of our participants align with broader systemic issues identified by Law et al.,^7^ who found “treatment inertia” and “healthcare disconnect” as central themes. Their finding that patients may live with suboptimal control rather than face treatment uncertainty resonates with our participants’ struggles with medication adherence due to side effects and fragmented care experiences.

The prolonged diagnostic journeys discussed by some participants, often involving misdiagnosis with psychiatric conditions, reflect broader patterns in rare neurological conditions. Similar diagnostic delays have been associated with increased psychological distress and poorer patient-provider relationships in rare epilepsies^22^ and neuromuscular disorders more broadly.^23^

This adaptation also involved accepting that the emotional journey following diagnosis is not a one-off process but a continuous one, shaped by the recognition that life may never fully return to how it was before. This echoes both Law et al.'s^7^ emphasis on adaptation as continuous rather than time-limited, and Keer-Keer et al.'s^15^ phenomenological finding that uncertainty represents a constant reminder that disease is present, even during remission.

Several participants expressed feeling inadequately supported during the process of coming to terms with their diagnosis, especially after lengthy and often complicated diagnostic journeys. Mistry and Simpson^24^ suggest that psychological interventions - particularly acceptance-based and mindfulness therapies - may support emotional adjustment following diagnosis of Motor Neurone Disease (MND). These approaches have also shown promise in helping people with cancer manage the psychological impact of diagnosis and ongoing illness,^25^ and may likewise hold potential for supporting individuals with MG. Despite this, psychological support is not yet routinely offered as part of the diagnostic process for MG, although our findings suggest that integrating such support could be valuable. In addition, participants highlighted a need for clearer communication around treatment expectations. Better information and continuity in care - such as minimising unexpected changes in care teams - could help reduce feelings of unpredictability and build trust between patients and medical professionals.

### Social and identity disruption

The social and identity disruption experienced by the majority of our participants reflects fundamental changes in how individuals relate to others and perceive themselves. This echoed Keer-Keer et al.^15^ in identifying how uncertainty leads to psychosocial distress and shifts in the relationship between the self and others.

Similar to what was identified by Law et al.,^7^ a few participants attributed platonic and romantic relationship breakdowns to MG, although we didn’t specifically ask about this and it was unclear which aspects of the illness contributed to these difficulties. The energy required to manage uncertainty often hindered social engagement, leading to feelings of isolation. Many participants shared difficulties in maintaining employment, reflecting the challenge of full-time work against the backdrop of residual symptoms and fatigue. This observation mirrors the findings of Guastafierro et al.,^26^ who reported notably low employment rates among individuals with MG. The loss of career aspirations and functional independence reflects profound disruptions to life trajectory that extend beyond symptom management, fundamentally altering individuals’ sense of identity and social role.

These psychosocial challenges closely mirror experiences reported in multiple sclerosis (MS), including distress tied to disease unpredictability and identity disruption,^27^ as well as employment difficulties associated with psychological distress and work instability.^28^ However, MG's day-to-day and minute-to-minute variability in muscle strength creates unique adaptation demands, necessitating continual social recalibration that distinguishes it from conditions with more predictable progression patterns.

Participants described how facial weakness hindered their ability to convey emotions effectively, disrupting both their sense of identity and the quality of social interaction. This impaired emotional expression appeared to contribute to a sense of inauthenticity in how they were perceived. Such findings align with existing research showing that individuals with MG exhibit reduced emotional expressiveness and diminished ability to recognize and express emotions^29^ compared to healthy controls. Moreover, similar psychosocial challenges are observed in related neuromuscular conditions, such as facioscapulohumeral muscular dystrophy (FSHD), where facial weakness is associated with poorer social functioning and elevated fear of negative evaluation.^30^

### Emotional and psychological impact

The emotional and psychological impact theme encompasses a complex interplay of symptoms that may or may not be linked to physical symptoms, potentially representing both clinical and subclinical psychiatric comorbidity. Whilst three of our participants had formal depression diagnoses, many others described self-reported low mood as an inevitable consequence of living with MG. This could suggest potentially unmet mental health needs that may not be captured through routine clinical assessment. Although one participant reported accessing CBT and another antidepressants, others described managing their emotional distress alone or relying on family members for support. Although not the primary focus of our study, participants’ accounts of emotional reliance on family members and the interpersonal consequences of their illness underscore the broader impact of MG on family systems. This aligns with a substantial body of research in other chronic neurological conditions, which documents significant caregiver burden. For example, in MS, caregiver distress has been strongly associated with patient cognitive and psychiatric symptoms, even when controlling for physical disability.^31^ In MND, caregiver burden has been shown to intensify as the disease progresses and may contribute to increased patient depression.^32^ These findings suggest that similar reciprocal dynamics may also be present in MG, particularly as emotional strain and physical dependency intersect in long-term care relationships.^33^ Future research should explore these bidirectional relationships within the MG population to better understand how psychological wellbeing is shaped not only by individual illness experiences but also by relational and family system dynamics.

The emotional burden described by participants also included frequent experiences of other negative emotions such as anxiety, frustration and embarrassment in relation to day-to-day life with MG. This pattern mirrors key emotions from Law et al.'s^7^ identification of anxiety, frustration, guilt, anger, loneliness and depression as central to the MG experience. The fact that all participants mentioned experiencing negative emotions day to day related to their MG experience, despite symptom stability, suggests that emotional support needs may persist independently of acute symptom management.

Further exploration is warranted into the anxiety associated with traumatic respiratory crises, which two participants described as particularly distressing events. Both reported ongoing anxiety rooted in the fear of recurrence, including avoidant behaviours which impacted their lives such as avoiding physical contact to not get colds. These experiences underscore the potential value of trauma-informed care, a framework that has been emphasised in other conditions involving respiratory crises as an important component of treatment following such events.^34^

In addition, cognitive difficulties such as mental fogginess and memory issues led some participants to experience a loss of confidence in their own thinking, contributing to identity disruption and a diminished sense of vibrancy. Our participants provided rich phenomenological descriptions of this experience, characterising it as distinct from physical fatigue and encompassing memory difficulties, concentration problems, and subjective mental inefficiency. While it remains unclear whether these issues stem from the disease itself, treatment side effects, or other factors, our findings suggest a likely multifactorial origin. This is consistent with previous research indicating that people with MG report high levels of self-assessed fatigue, even when objective measures do not reflect this.^35^ Further research is needed to better understand the lived experience of these cognitive and emotional challenges, and which elements of the condition drive them.

The relationship between effective MG treatment and psychological outcomes is complex. Andersen et al.'s^36^ randomised controlled trial demonstrated that eculizumab significantly improved fatigue scores in correlation with improvements in other MG-specific outcomes, suggesting that psychological symptoms like fatigue should improve when highly effective physical treatment achieves dramatic symptom control. However, our findings suggest that significant psychosocial challenges persist even during periods of symptom stability and medical management. While our participants may have had varying levels of residual physical impairment despite their stability, the persistence of complex psychosocial challenges during stable periods indicates these are not solely related to acute symptom fluctuation, crisis episodes, or lack of medical treatment. This suggests that psychosocial support may be needed even when patients are clinically stable and receiving appropriate treatment.

### Impact of uncertainty

Mishel's Uncertainty in Illness Theory^37^ provides a framework for understanding how people experience uncertainty when they struggle to understand or predict their illness because symptoms, treatments, or disease progression don't follow clear, predictable patterns. The theory identifies four forms of uncertainty: ambiguity (unclear symptom patterns), complexity (overwhelming treatment complexity), inconsistency (incongruence between expected and experienced symptoms), and unpredictability (uncertain illness trajectory). Our findings richly illustrate all four dimensions within the MG experience, with particularly profound impacts stemming from symptom unpredictability and treatment complexity. The identified psychosocial burdens reflect the theoretical view that illness-related uncertainty can deeply impair adaptation. Prior research corroborates these observations, suggesting that uncertainty is central across chronic illnesses. For instance, Camdessanché et al.^21^ found that the fluctuating and unpredictable nature of MG hindered long-term planning and prompted patients to adopt withdrawal coping strategies.

The unpredictability of muscle strength necessitates continual adaptation, echoing Keer-Keer et al.,^15^ who described uncertainty as a pervasive experience across the MG disease trajectory. This pervasive uncertainty complicates efforts to cope and adapt, placing strain on relationships, identity, and emotional wellbeing. It also amplifies psychological difficulties, including persistent fears of re-encountering previously distressing symptoms or crises. Importantly, the psychosocial challenges borne of uncertainty in the lived experience appears to persist even when physical symptoms are stable, underscoring the need for integrated psychological support within MG care pathways.

Our analysis suggests that whilst uncertainty is recognised as a key known part of chronic illness in general, in MG has several distinct characteristics differentiating it from other chronic conditions. The day to day and minute-to-minute variability in muscle strength creates a unique form of embodied uncertainty that distinguishes MG from conditions with more predictable progression patterns. This uncertainty appears to create a cyclical pattern where stress-induced symptom exacerbation^38^ is perpetuated by ongoing uncertainty, contributing to feedback loops that reinforce the complexity of the psychosocial burden. Unlike progressive conditions such as motor neurone disease, where psychological adaptation often centres on anticipatory grief and end-of-life concerns,^39^ MG presents a distinct psychological terrain where fluctuating course creates ongoing uncertainty about daily capabilities, yet simultaneously preserves hope for improvement. Uncertainty, in this sense, both drives and is compounded by the challenges of living with MG, reinforcing the complexity of the psychosocial burden.

### The role of psychosocial and therapeutic support

Our findings suggest a need for targeted psychotherapeutic and social support to address the complex psychosocial challenges of MG. Given uncertainty's role as a meta-theme, interventions should specifically address the unpredictable nature of MG and teach adaptive coping strategies for managing day-to-day symptom variability and future concerns. This may include the integration of self-management strategies grounded in promoting perceived self-efficacy,^40^ which can empower individuals to manage the chronic uncertainty of the condition. This supports the call by Tigner et al.^41^ for structured self-management plans tailored to the needs of MG patients, fostering collaboration with healthcare teams while facilitating goal management, symptom monitoring, and skill development. Such strategies could enhance social engagement and address the multifaceted psychosocial impacts of the disease.

Additionally, cognitive behavioural therapy (CBT) could improve emotional resilience, as demonstrated in conditions like cerebral palsy^42^ and MND.^43^ Given the physical limitations and social isolation experienced by MG patients, structured peer support programs (especially those delivered online) could help counteract social withdrawal by creating a safe space to share practical coping strategies and lived experience. Systematic reviews suggest that online peer support can promote social support, self-efficacy, social participation, and health-directed behaviours, which helps individuals manage chronic conditions more effectively and feel less isolated.^44^

It is important to note that there appears to be a clear discrepancy between the psychosocial needs of MG patients and the treatment currently available. Healthcare providers should recognise that achieving symptom stability does not automatically resolve the psychological and social impacts of living with MG, and that routine consideration of psychosocial functioning may be warranted as part of comprehensive MG care.

### Limitations and future research directions

Overall, this study offers rich qualitative insights into a frequently overlooked dimension of the MG experience, contributing to the growing body of research aimed at more accurately capturing quality-of-life determinants and identifying unmet needs in clinical practice. Our findings suggest that comprehensive MG care must extend beyond managing physical symptoms to also address the persistent psychosocial burdens patients face. However, several limitations must be acknowledged when interpreting these findings.

Most notably, the small sample size (n = 8) limits the generalisability of findings, particularly given the clinical heterogeneity of MG. This limitation reflects both the rarity of MG and the difficulty of identifying clinically characterised individuals for qualitative interviews. These recruitment challenges highlight the value of conducting secondary analyses to maximise insights from existing datasets.

Although more individuals were initially interviewed, the final sample was reduced due to the exploratory nature of the original research aims meaning clinical characteristics were not collected for all participants, one participant choosing to withdraw their data, and the exclusion of individuals with CMS to ensure diagnostic specificity. While this led to a small final sample, the presence of shared themes across participants suggests certain psychosocial challenges may be common across age groups and illness durations. Table 1 provides individual-level data, which shows variation in participants’ demographics, including a broad age range (23–78 years) and time since diagnosis (2–20 + years). These factors may have influenced participants’ perceptions, as adaptation strategies likely differ between younger versus older individuals and those newly diagnosed versus long-term patients. However, the consistency of some themes across this varied sample offers preliminary evidence that these challenges may transcend specific subgroups, though we cannot exclude the possibility that certain demographic or clinical factors disproportionately influenced the themes identified.

Participant characteristics such as age, gender, disease duration, and comorbidities may meaningfully influence the psychosocial burden of MG. Comorbid depression is highly prevalent, with a recent meta-analysis estimating a pooled prevalence of 36% (95% CI: 28–45%) among people with MG.^10^ In our sample, three participants had documented depression, with one taking antidepressant medication and another undergoing cognitive–behavioural therapy. The presence and treatment of depression may have amplified the emotional burden described by these individuals, influencing how they narrated experiences of fatigue, demoralisation, and social withdrawal. Gender also appears relevant: a single-centre cohort study of 165 patients found that women reported significantly poorer disease-specific quality of life than men (mean MG-QoL15: 19.7 vs. 13.0; p = 0.024; Twork et al., 2023), consistent with other reports of gender-related differences (Kim et al., 2020). Age and disease duration may further modulate psychosocial impacts through their effects on employment, social roles, comorbidity burden, and coping resources. In our data, while no systematic subgroup differences were evident, younger participants more often reflected on the impact of MG on education and peer relationships, whereas older participants emphasised difficulties in sustaining activities they once enjoyed and uncertainty about whether decline was attributable to MG or to ageing. These observations remain provisional given the small and heterogeneous sample, but they align with prior evidence that psychosocial concerns may shift across the life course. Future research with larger, clinically well-characterised cohorts is warranted to examine systematically how demographic and clinical variables (particularly comorbid mood disorders) shape the lived experience of MG.

Another limitation is the absence of a control group. Including a comparator group - such as individuals with CMS, other chronic illnesses, or healthy controls - was not feasible due to small numbers and lack of clinical characterisation, precluding subgroup analysis. As a result, it is difficult to determine the extent to which the psychosocial challenges identified are specific to MG versus common across chronic illness more broadly.

Secondary analysis also imposed constraints related to the original study design. Although participants were required to be clinically stable for at least three months (defined as no major symptom fluctuation, hospital admission, or treatment change), residual symptom severity was not systematically assessed using standardised measures such as the MG-ADL or MG-QoL-15r. This limits the clinical characterisation of our sample and introduces the possibility that ongoing psychosocial difficulties may reflect significant, albeit stable, physical impairments rather than separate psychological or social phenomena. Thus, while our findings indicate that psychosocial challenges persist in the absence of acute symptom change, it remains possible that participants still experienced substantial physical burden. We acknowledge that a three-month stability period may still include patients in earlier phases of disease fluctuation, and future work may benefit from a longer period (e.g., ≥ 12 months) to ensure enduring clinical stability.

Additionally, the primary focus of the original interviews was on daily experiences with physical symptoms. As a result, psychological and social impacts may have been underexplored. Specific probing about mental health needs or support experiences may have revealed further, or different, psychosocial challenges. Importantly, our participants had relatively stable disease and were engaged with specialist care services. Individuals with more volatile symptom profiles, shorter time since diagnosis, or less access to specialist care may experience even greater unmet psychosocial needs.

Several directions for future research arise from our findings. The persistence of psychosocial challenges despite clinical stability highlights the need for longitudinal studies examining how psychological and social experiences evolve across different phases of MG. Including a control group would help distinguish MG-specific needs from general chronic illness experiences. Larger-scale, survey-based studies could also explore which psychosocial domains are most commonly affected in MG and identify which subgroups are most at risk. The cognitive difficulties reported by participants, such as mental fatigue and problems with planning, warrant dedicated investigation using both objective neuropsychological assessments and qualitative methods to capture lived experiences. Furthermore, given the prominent theme of energy management and daily planning burden, evaluating structured self-management interventions tailored to MG may be beneficial. Finally, future research should also explore the reciprocal relationship between patient psychological wellbeing and caregiver burden, with the aim of developing more family-centred support models.

## Conclusion

This exploratory study highlights persistent psychological and social challenges in MG patients, even when symptom stability is achieved. The findings, echoing convergent evidence from international research, underscore the need for integrating psychological and social support into patient-centred MG care. Healthcare providers should recognise that symptom stability does not equate to psychosocial wellbeing and should consider routine psychosocial assessment as standard practice in comprehensive MG care. Future research should explore whether targeted psychotherapeutic interventions can improve quality of life for MG patients, with particular focus on managing uncertainty and enhancing psychosocial adaptation during periods of medical stability.

## Abbreviations

AChR: Acetylcholine Receptor
ALS: Amyotrophic Lateral Sclerosis
CBT: Cognitive Behavioural Therapy
CMS: Congenital Myasthenic Syndromes
MG: Myasthenia Gravis
MGFA: Myasthenia Gravis Foundation of America
MND: Motor Neurone Disease
MuSK: Muscle-Specific Kinase
